# Norwegian general practitioners’ perceptions of their depression care – a national survey

**DOI:** 10.1186/s12875-024-02434-0

**Published:** 2024-05-24

**Authors:** Ina Grung, Stefan Hjørleifsson, Norman Anderssen, Berit Bringedal, Sabine Ruths, Øystein Hetlevik

**Affiliations:** 1 Research Unit for General Practice, NORCE Norwegian Research Centre, Bergen, Norway; 2 Department of Global Public Health and Primary Care, University of Bergen, Bergen, Norway; 3 Department of Psychosocial Science, University of Bergen, Bergen, Norway; 4 Institute for Studies of the Medical Profession, Oslo, Norway

**Keywords:** Depression, General practitioners, Talking therapy, Primary health care

## Abstract

**Background:**

The General Practitioner (GP) is often the first professional contact for patients with depression. Depression care constitutes a substantial part of GPs’ workload.

**Objective:**

To assess how GPs experience their patients’ expectations and their own provision of depression care; further, how their depression care was associated with doctor- and practice-characteristics.

**Methods:**

A cross-sectional questionnaire study about depression care in general practice among the GPs in the Norwegian Physician Survey of 2021.

**Results:**

Of the 221 responding GPs, 50% were female and 70% agreed to have constant time pressure due to workload. The GPs believed that patients with depression were interested in their professional assessment (87.2%) and saw them as providers of talking therapy (76,9%). Still, 77,8% of the GPs thought the patients expected a referral. Talking therapy was commonly provided (79.6%) along with consultations of more than 30 min (80.4%). The youngest age group and GPs with shorter patient lists spent more time. Most GPs (92.3%) considered their help to be of great benefit for depressed patients. However, one-fourth of the GPs did not feel competent in providing talking therapy, less frequently reported by the GPs aged 40–54 years.

**Conclusions:**

Talking therapy is commonly provided by GPs. However, there is a need to investigate what GP talking therapy implies, and to strengthen GP skills in this regard. Overall, the GPs experience their depression care to be useful for their patients, and do not de-prioritize this although they experience workload pressure.

**Supplementary Information:**

The online version contains supplementary material available at 10.1186/s12875-024-02434-0.

## Key messages

GPs provide talking therapy and long consultations for patients with depression, even when experiencing workload pressure.

GPs believe that their help is of great benefit to patients with depression.

One out of four GPs does not feel competent in providing talking therapy.

**Supplementary Information:**

The online version contains supplementary material available at 10.1186/s12875-024-02434-0.

## Background

Depression is one of the three leading causes of years lived with disability globally [1]. The World Health Organization (WHO) claims that integrating mental health services into primary care is the best way to provide good and equitable care for mental health problems [2]. In countries with a strong primary health care, such as Norway, guidelines for the management of depression recommend psychological treatment in primary care as the first intervention in mild to moderate cases [3–5]. In such countries, the general practitioner (GP) is usually the first contact for all kinds of health problems and often represents continuity of care over time and across different health problems [6]. GPs thus have an important role in diagnosing and treating patients with depression [7, 8].

In Norway, 11% of GP consultations in 2022 was reported to concern psychological problems, with depression being the most common diagnosis. Approximately 4% of the population aged 15–64 consult their GP for depression each year [9].

GPs’ depression care can consist of talking therapy; from supportive dialogue to more structured therapy, and prescription of antidepressant medications. GPs also act as gatekeepers to secondary care and refer patients when necessary.

A meta-analysis of randomized controlled trials of brief psychological therapies in primary care indicated that brief psychological therapies by GPs were effective, and emphasized the therapeutic value of a well-functioning GP-patient relationship [10]. This is partly supported by a survey among patients recruited in GPs’ waiting rooms in Norway, where 59% of the 770 participants who had experienced depression care preferred talking therapy by GP to other treatment options [11]. However, 60% reported that they would prefer a referral to a specialist, indicating that many patients do not expect GPs to manage depression cases.

A meta-analytic review across different settings yielded a 70% greater patient preference for psychological treatment as the first treatment option for depression, compared to pharmacological treatment [12].

In recent decades, mental health care has been strengthened in Norwegian primary care [13]. Initially, this was directed towards patients with chronic diseases, yet recently also with treatment for common mental disorders. From 2020, community psychologists became a mandatory part of the psychosocial care in the municipalities [13]. However, this is still not fully implemented, community psychologists are not necessarily involved in clinical services and their role in relation to general practice is not yet clear. Public information communicates that the GP can help with mild symptoms [14], but a main expectation in the public can be that the GP first of all will help you to get in touch with other services and that mental health problems do not belong in the GPs offices.

In sum, GPs play an important role in providing depression care in Norway, in accordance with recommendations from the health authorities. There is, however, limited knowledge of their experiences and opinions on this part of their work. The aim of this study was to assess how GPs experience their patients’ expectations and their own provision of depression care; further, how their depression care was associated with doctor- and practice-characteristics.

## Methods

### Setting

All residents of Norway have access to public health services through the National Insurance Scheme and are entitled to their own GP according to the Regular General Practitioner scheme. Each GP has a fixed list of patients. In 2020, the average list size was 1096 [15]. In the general population, 71% have at least one contact per year with their GP [16]. Norwegian GPs meet an unselected patient population, provide comprehensive care for a broad range of health issues, and act as gatekeepers to specialist healthcare.

### Study population

The Norwegian Physician Survey is a biennial survey of a representative panel of doctors working in Norway (*N* = 2316). Its representativeness is compared with the Norwegian Medical Association’s membership list, which includes 94% of all doctors working in Norway. The panel as a whole shows a similar distribution to the general population of physicians in terms of age, gender, and proportions working in primary care, public and private specialist care.

In the current study, only GPs are included. We conducted a cross-sectional study on depression care in general practice among GPs in the Norwegian Physician Survey of 2021. In the subsample of GPs (*N* = 221) in this study, we found the distribution of age and list length to be in line with the national sample of GPs, while there was a slightly larger proportion of female GPs (50% vs. 45%) and employed GPs (19% vs. 15%) in the study compared to the national sample. We do not know the exact number of GPs in the panel. However, it is reasonable to assume a similar response rate for GPs as for the rest of the panel.

### Questionnaire

In December 2020, a postal and electronic questionnaire was sent to the panel, with two reminders in the first months of 2021 (Supplementary file 1).

The questionnaire collected information on doctors’ demographic data, working conditions and working hours, prioritization and health, ethical dilemmas, pandemic work situation, job satisfaction and health and workload.

The section concerning depression care was to be answered by GPs only. The questions were developed by the authors and informed by contributions from the larger research project “The regular general practitioner scheme: integrated and equitable pathways of depression care, facilitating work participation” (The Norwegian GP-DEP study) [17], which the current study is part of. The present study is mainly based on 13 statements under the heading “When a patient seeks help from me due to possible depression, the following applies”. Additionally, we used three questions regarding the GPs’ views on municipal mental health care, one question regarding workload, and demographic data reported by the participants.

The GPs were asked to score each of the statements on a 4-point Likert scale from 1 to 4, with the alternatives “very often”, “often”, “rarely”, “never” and “does not apply” or “partially agree”, “completely agree”, “quite disagree”, “completely disagree” and “does not apply”.

The questionnaire was piloted by five GPs, and their comments were used to improve the phrasing.

### Statistical analysis

Descriptive statistics was used to examine the distribution of GP and practice characteristics, and the responses to statements about depression care. We then dichotomized the responses, grouping together “very often” and “often” in one category, and “rarely” and “never” in another. We used Chi-square statistics to test for differences in the distribution of the dichotomized responses. Significance level was set to *p* < 0.05. Stata/SE version 18.0 (Stata Statistical Software) was used for statistical analyses.

## Results

Altogether 1639 physicians (71% of all panelists) responded to the full survey. A total of 221 GPs answered one or more of the GP-only questions about depression care, making up the current study population, Table 1. Half of the respondents were female, and the GPs were quite evenly distributed in the age groups below 40, between 40 and 55 years and above 55 years. One out of three GPs had graduated outside Norway. Most were self-employed (73.8%) and worked in group practices (98.5%). Most GPs (70%) agreed or partly agreed that they had a constant time pressure due to workload.

**Table 1:** Demographic data, practice characteristics, and self-reported working conditions for participating GPs (*N* = 221)

	*N*	%
**GP characteristics**		
*Gender*		
Female	110	50.0
Male	110	50.0
*Age*		
<40 years	82	37.3
40–54 years	66	30.0
55 + years	72	32.7
Missing	1	
*Country of graduation from medical school*		
Norway	144	66.1
Outside of Norway	74	33.9
Missing	3	
**Practice characteristics**		
*Form of employment*		
Self-employed	163	73.8
Employee	42	19.0
Other	16	7.2
*Length of patient list*		
<900	66	33.5
900–1199	65	33.0
>1200	66	33.5
Missing	28	
*Practice type*		
Group practice	200	98.5
Solo practice	3	1.5
Missing	18	
**Self-reported workload**:		
*“I am experiencing constant time pressure due to workload”*		
Completely agree		40.2
Partially agree		38.8
Partially disagree		16.4
Completely disagree		4.6

The first four statements in Table 2 can be seen as GPs’ assumptions regarding patient preferences and expectations when patients with depression consult them. Most GPs considered the patients often or very often to be interested in their professional assessment, regarding the GP as a provider of talking therapy, and seldom or never wanting medication as the only treatment. Yet, three out of four GPs reported that they often or very often believed that the patient expected a referral.

**Table 2:** Responses to statements about depression care introduced by the phrase “When a patient seeks help from me due to possible depression, the following applies”

	*N*	Very often%	Often %	Rarely %	Never %	Not applicable %
The patient considers me as a provider of talking therapy	221	26.7	50.2	20.3	1.4	1.4
The patient is interested in my professional assessment	220	33.6	53.6	11.8	0.5	0.5
The patient wants antidepressants as the only treatment	220	0	9.1	79.5	10	1.4
The patient expects to be referred to a psychologist or psychiatrist	221	24.0	53.8	19.5	0.5	2.2
I conduct talking therapy with the patient	221	27.6	52	16,7	2.3	1.4
I spend more than 30 min in the consultation	220	29.5	50.9	18.2	0.9	0.5
Mapping the patient’s problems is more important than determining whether the criteria for the depression diagnosis are met	220	31.8	58.6	7.7	0.5	1.4
The patient’s preferences are important for the kind of help I offer	221	16.3	66.5	14.9	1.4	0.9
I recommend antidepressants, but the patient does not want this	221	0.5	19.4	73.3	4.1	2.7
I provide antidepressants along with talking therapy	220	9.5	61.8	25.0	1.4	2.3
My help is of great benefit to the patient	220	20.0	72.3	6.8	0	0.9
Offering talking therapy is beyond my competence	221	5.9	21.3	45.7	24.9	2.2
I am the only healthcare professional the patient sees	219	4.1	45.0	46.3	4.1	0.5

The next six statements regard provision of depression care, where most GPs (79.6%) reported to conduct talking therapy with their depressed patients often or very often. Further, 80.4% stated that they often or very often spent more than 30 min on these consultations. The majority (90.7%) considered mapping the patient’s problems often or very often as more important than determining whether the depression diagnosis criteria were fulfilled. Approximately 70% reported that when they prescribed antidepressants, this was often or very often combined with talking therapy.

The last three statements can be seen as a self-evaluation of their depression care, where 92.3% of the GPs believed that their help often or very often was of great benefit to their patients with depression. Most GPs (70,6%) stated that they felt competent in providing talking therapy. Half of the GPs responded that they often or very often were the only health care worker the patient was in contact with.

Spending more than 30 min on a consultation with a depressed patient was significantly more commonly reported by GPs younger than 40 years compared to those 40 years or older (Table 3). Further, long consultations were significantly more commonly reported by GPs with the shortest patient lists (size < 900) than those with longer lists. Considering talking therapy to very often/often to be beyond one’s competence was less frequently reported by the GPs aged 40–54 years. Self-employed GPs stated significantly more commonly to provide talking therapy than those employed.

**Table 3:** Responses to statements about conducting talking therapy, time used in the consultations and talking therapy-skills, with distribution regarding GP- and practice characteristics (proportions, with chi square test)

	I conduct talking therapy					I spend more than 30 min on the consultation					Offering talking therapy is outside my competence			
	N	Very often/ often	Rarely/ Never	p		N	Very often/often	Rarely/ Never	p		N	Very often/ often	Rarely/ never	P
		%	%				%	%				%	%	
Total	221	79.6	19.0			220	80.4	19.1			221	27.2	70.6	
*Gender*				0.513					0.057					0.780
Female	108	82.4	17.6			109	86.2	13.8			106	28.3	71.7	
Male	107	78.9	21.1			109	76.1	23.8			109	26.6	73.4	
*Age*				0.064					0.013					< 0.001
<40 years	81	74.1	25.9			81	88.9	11.1			81	43.2	56.8	
40–54 years	66	89.4	10.6			66	83.3	16.7			65	10.8	89.2	
55 years or more	70	80	20			71	70.4	29.6			69	24.6	75.4	
*Country of graduation*				0.211					0.727					0.760
Norway	141	78	22			142	81.7	18.3			141	28.4	71.6	
Outside of Norway	74	85.1	14.9			74	79.7	20.3			72	26.4	73.6	
*Form of employment*				0.001					0.263					0.420
Self-employed	163	86.5	13.5			162	80.2	19.8			161	25.5	74.5	
Employee	41	63.4	36.6			41	87.8	12.2			41	31.7	68.3	
*Length of patient list*				0.560					0.001					0.270
<900	66	80.3	19.7			66	90.9	9.1			65	27.7	72.3	
900–1199	65	80	20			65	86.1	13.9			65	32.3	67.7	
>1200	66	86.4	13.6			65	66.1	33.9			65	20	80	
*Practice*				0.409					0.407					0.380
Solo practice	3	100	0			3	100	0			2	0	100	
More than one GP	199	81.4	18.3			198	81.3	18.7			198	27.3	72.3	
*Response to statement: Constant time pressure due to workload*				*0.613*					*0.080*					*0.873*
Completely disagree	9	88.9	11.1			10	60.0	40.0			10	20.0	80.0	
Disagree	34	73.5	26.5			35	77.1	22.9			34	23.5	76.5	
Agree	85	80.0	29.0			85	78.8	21.2			83	28.9	71.1	
Completely agree	88	83.0	17.0			87	88.5	11.5			87	28.7	71.3	

When assessing internal associations in the responses, we found a non-significant trend towards more long consultations with higher total workload. Among the GPs reporting to carry out talking therapy often or very often,15% reported that they often regarded this to be outside their competence (not shown in tables).

The GPs were overall positive towards a reinforcement of the municipal mental health services with first line psychologist, and to link them to the GP practices (Table 4). However, the GPs had diverging views on taking on the role of coordinator for the use of other municipal mental health services.

**Table 4:** GPs’ response to the statement: “Based on your experience, how much do you agree or disagree with the following statements about municipal initiatives”

	*N*	Completely agree	Partially agree	Quite disagree	Completely disagree	Doesn`t apply
Municipal services for treatment by a psychologist without a referral should be expanded	219	65.3	20.1	6.4	5.9	2.3
Psychologists in the municipalities should be linked to GP practices	219	40.7	30.1	19.2	5.9	4.1
The GP should have a coordinating role for all mental health services	219	14.2	23.3	37.0	21.9	3.6

## Discussion

### Statement of principal findings

In this questionnaire study of a representative sample of 221 Norwegian GPs regarding their depression care, we found that that most GPs believed their depressed patients often were interested in their professional assessment and considered them as providers of talking therapy. Four out of five reported to provide talking therapy often or very often. Still, they were often under the impression that patients expected a referral to a psychologist or a psychiatrist. Three out of four GPs reported that they often or very often felt competent in talking therapy. More than 85% wanted the recent national implementation of community psychologists to be expanded.

### Comparison with existing literature

In this study, most GPs felt that patients seeking help for depression were interested in their professional assessment and considered them providers of talking therapy. However, 78% believed that patients often expected a referral. This apparent contradiction is in line with a survey about patients’ preferences in case of a future depression. While 61% would prefer talking therapy with their GP, 53% would prefer a referral, indicating some wanting both [11]. These seemingly inconsistent data from GPs and patients may imply that there is a potential for a productive relationship between the GP and depressed patients – while many patients want a referral, they are also open for talking therapy with their GP.

Our study indicates that Norwegian GPs often provide talking therapy when patients consult them for depression, and that GPs often spend more time in these consultations than the usual 15–20 min in typical Norwegian GP consultations. This finding is supported by a national register study in Norway, which documents that both GP talking therapy and long consultations with depressed patients is commonly provided; in nearly half the consultations regarding depression more than 20 min were spent [18].

There was also a trend towards more often spending extra time when the GPs experienced more time pressure in their practice. We cannot know whether the GPs are experiencing being busy *because* they spend much time with their patients – or if they just continue to spend time with their patients even when they are experiencing time pressure. Anyhow, our findings suggest that patients with depression and a need for more time is not de-prioritized, which is in line with a Dutch study [19], where 2095 videotaped consultations were analysed: The findings indicate that GPs are aware of patients’ psychological problems even when they are busy and have many patients waiting. However, in our study, long consultations were less often reported by GPs with longer patient lists.

Most GPs found it more important to map the patient’s problems than to determine whether the criteria for a depression diagnosis were met. This resonates well with the findings from earlier studies [20, 21]. In previous literature the GPs’ main focus on the patients’ problems and their life context is presented as a more patient-centered approach [22]. However, other research suggests that depression is less frequently diagnosed when patients have difficult lives – implying that treatment for depression can be harder to get for the less privileged, i.e., the “inverse care law” [23].

A Norwegian registry-study found that about 25% of the patients with a new depression diagnosis in general practice were prescribed antidepressants [18]. We did not include the frequency of prescriptions in our study. But the GPs rarely considered patients to want medication as the only form of treatment, and they rarely experienced patients refusing the antidepressants they suggested. This indicates that GPs, in their own experience, do not try to persuade patients with depression to take medications, which intriguingly seems to be at odds with the findings of a qualitative questionnaire study [24] where the patients felt they were “pushed towards” medication by their GP. In the present study, the GPs experience themselves as patient-oriented and aware of their patients’ preferences when it comes to treatment for depression. When medication was prescribed, this was mostly done together with talking therapy, in line with recommendations [3] and patient preferences [24].

More than 90% of the GPs in the study believed that their help often or very often was of great benefit to the patients. As this was a wide-ranging question covering all kinds of help, it might be related to a patient-centered approach; a majority reported that mapping the patients’ problems was more important than finding out whether the depression diagnosis criteria were met. Continuity of care is seen as a core value in general practice and has in numerous studies been linked to positive outcomes of therapy in general [25]. Continuity of care may foster a therapeutic relationship, which has been reported to be a crucial factor in the outcome of psychological therapy [26].

Half of the GPs in the survey reported that they were often or very often the only health care worker the patient was in contact with. This is in line with earlier findings [27]. Traditionally, GPs are the first line service, treating patients as well as being coordinators and gate-keepers to specialist health care [28]. The GPs seem open to sharing their role in primary care, as most GPs agreed that low-threshold municipal psychology services without referral should be built out. A large proportion of the GPs would also welcome this service to be affiliated with the GP-offices. In other European countries mental health nurses and psychologists are linked to the GP practices [29, 30], and a more team-based GP service in mental health [31], including depression care, seems to have support among the GPs.

### Implications for research and practice

The content and methods used in GPs’ talking therapy is understudied. Talking therapy provided by GPs includes supportive talk, counselling, or structured psychotherapeutic methods such as cognitive-behavioral therapy [5]; still, their approach is most likely less structured than therapies offered by psychiatrists and clinical psychologists. There is a need for further studies to get more insight into how GPs provide talking therapy to depressed patients, and the effectiveness of talking therapy provided by GPs.

Further, there is a need for providing GPs with more training in talking therapy, since one out of four GPs report that they lack the skills.

It also seems necessary to consider available resources, so that working conditions for GPs allow time to treat patients with depression, since GPs already see a lot of patients with depression and many of them provide talking therapy and show an interest in this. GPs who know their patients over time and also deal with somatic conditions commonly related to depression, could have a potential for an expanded role in depression care, provided resources are transferred to this service.

The new low-threshold mental health services in Norway are a supplement to GPs in depression care. They are most often established independently, and not linked to GP practices, where most of the first-line depression care currently takes place. There is a need for further research into how the mental health service in primary care is best organized, for the benefit of patients and with respect to efficiency.

### Strengths and limitations

A strength of this study is that the survey employs a panel of GPs who have agreed to participate in regular surveys and the panel at large is representative of Norwegian physicians [32]. The sample of GPs responding in this study seems representative of the GP population in Norway, as it aligns well with the distribution of age, gender, and list length, although with a somewhat higher proportion of employed GPs.

A limitation of study is that the GPs may have based their responses on their experiences with patients with varying severity of depression or depressive symptoms, and they may have interpreted some of the central terms of the study in different ways. For example, the term “talking therapy” is not clearly defined and GPs may have different understandings of when they provide this treatment. This may have led to both under- and overreporting, depending on their understanding of these terms. However, the Norwegian GP reimbursement coding system uses the term “Therapeutic conversation: Talking therapy by GP at least 15 minutes long with patients with psychological problems.The conversation must deviate from a normal conversation on medical issues and have a therapeutic character” [33], thus it is a term GPs in Norway relate to.

This study is based solely on GP-reported data, which may be biased, uncertain in which direction. However, when investigating experiences and attitudes, this is known to be a preferred and plausible method.

## Conclusions

Talking therapy is commonly provided by GPs in Norway. There is a need for further research into what talking therapy implies, also there is a need for building up GPs` competence in this regard.

GPs experience their depression care to be useful for their patients and do not de-prioritize this despite their heavy workload.

### Electronic supplementary material

Below is the link to the electronic supplementary material.

Supplementary Material 1

## Data Availability

No datasets were generated or analysed during the current study.
