# Supplementary material for: Norwegian general practitioners’ perceptions of their depression care – a national survey
Source: BMC Prim Care. 2024 May 24;25:184. doi: 10.1186/s12875-024-02434-0 (PMC11127373; doi:10.1186/s12875-024-02434-0)
Supplement: Supplementary file 1 — Supplementary Material 1 [file 12875_2024_2434_MOESM1_ESM.pdf]

Supplementary file 1

Paper: *Norwegian general practitioners' perceptions of their depression care – a national survey.*

The Norwegian Physician Survey is a biennial survey of a representative panel of doctors working in Norway. The following text is a translation from Norwegian to English, of the introduction and the parts of the questionnaire that were used in our article. The additional content is meant for other articles but may be provided on request.

…….

Dear colleague and member of the panel.

Welcome to this year's survey, the 15th since the beginning in 1994. The panel has approximately 2300 members and provide a representative sample of physicians working in Norway.

The last year have been imprinted with the Covid-19-pandemic and thus we will also raise questions on prioritization of patient treatment, working conditions and infection risk throughout this period.

This year's themes are:

A Working condition and working hours

B Prioritization

C Work situation and infection prevention during the Covid-19-pandemic

D The general practitioners' role in the treatment of patients with depression

E Satisfaction, health and workload

F Background information

The Survey contributes to that the knowledge base on work, health service design and health policy include the physicians' own experiences and views. Not all questions are suited for everyone, but we ask you to answer what you can.

You may answer electronically or on paper. We encourage you to use the electronic opportunity for responding, a solution we offer in cooperation with the University in Oslo. Write this link in the browser:

https://skjema.uio.no/legepanel-2021

It is important that the serial number at the top of this side is written twice in the electronic answer. Further instructions you will find in the survey.

The Survey is about working physicians with their main position in Norway. If you are not currently working, we ask you to respond from your latest workplace. If you are a full-time pensioner, write this in question A01.01 and return the questionnaire blank.

The response deadline is the 1st of February. If you do not respond electronically, please use the pre-stamped envelope for your response. If you have any questions or find something difficult, feel free to contact with:

Erlend Hem, mobil: 95730355 / erlend.hem@legeforeningen.no

Thank you for participating and contribute to a more solid knowledge on the physicians` professional life!

With regards,

Erlend Hem, Head of Institute

The Questions from D01 to D07 are TO BE ANSWERED BY GENERAL PRACTITIONERS ONLY.

If you are not a general practitioner, proceed to question E01 on page 12.

D The General Practitioner`s role in the treatment of patients with depression

This part of the questionnaire is about the follow-up of patients with depression.

**D01**	**When a patient seeks my help due to possible depression, the following applies:**	Very often	Often	Rarely	Never	Does not apply
D01.01	The patient is interested in my professional assessment	1	2	3	4	9
D01.02	The patient's preferences are important for the kind of help I offer	1	2	3	4	9
D01.03	The patient considers me as a provider of talking therapy	1	2	3	4	9
D01.04	The patient expects to be referred to a psychologist or psychiatrist	1	2	3	4	9
D01.05	Mapping the patient's problems is more important than determining whether the criteria for the depression diagnosis are met	1	2	3	4	9
D01.06	My help is of great benefit to the patient	1	2	3	4	9
D01.07	Offering talking therapy is beyond my competence	1	2	3	4	9
D01.08	I conduct talking therapy with the patient	1	2	3	4	9
D01.09	I spend more than 30 minutes in the consultation	1	2	3	4	9
D01.10	I recommend antidepressants, but the patient does not want this	1	2	3	4	9
D01.11	The patient wants antidepressants as the only treatment	1	2	3	4	9
D01.12	I provide antidepressants along with talking therapy	1	2	3	4	9
D01.13	I am the only healthcare professional the patient sees	1	2	3	4	9

………

During the last years, the municipalities have developed various services for patients with depression, such as «Rapid psychological health care» and other low threshold services without a need for referrals.

**D05**	**Based on your experience, how much do you agree or disagree to the following statements on such municipal initiatives:**	Completely agree	Quite agree	Quite disagree	Completely disagree	Does not apply
D05.06	Municipal services for treatment by a psychologist without a referral should be expanded	1	2	3	4	9
D05.07	Psychologists in the municipalities should be linked to GP practices	1	2	3	4	9
D05.08	The GP should have a coordinating role for all mental health services	1	2	3	4	9

………

E Satisfaction, health and workload

**E02**	**Correlation between effort and reward**	Completely agree	Quite agree	Quite disagree	Completely disagree	Does not apply
E02.01	I am experiencing a constant time pressure due to heavy workload	1	2	3	4	9
